# Supplementary material for: Predicting Subject Traits From Brain Spectral Signatures: An Application to Brain Ageing
Source: Hum Brain Mapp. 2024 Dec 20;45(18):e70096. doi: 10.1002/hbm.70096 (PMC11660765; doi:10.1002/hbm.70096)
Supplement: Supplementary file 1 — Data S1. Supporting Information. [file HBM-45-e70096-s001.pdf]

Predicting subject traits from brain spectral signatures: an application to brain ageing (SI)

Cecilia Jarne[a,b,c], Ben Griffin[e], Diego Vidaurre[c,1]

[a] *Departamento de Ciencia y Tecnologia de la Universidad Nacional de Quilmes, Bernal, Buenos Aires, Argentina*

[b] *CONICET, Buenos Aires, Argentina*

[c] *Center of Functionally Integrative Neuroscience, Department of Clinical Medicine, Aarhus University, Aarhus, Denmark*

[d] *Nuffield Department of Clinical Neurosciences, Oxford University, Oxford, UK*

[e] *Oxford Centre for Human Brain Activity, Department of Psychiatry, Oxford University, Oxford, UK*

**Country**	**Dataset sites**	**Synapse Name**	**N individuals (Female; Male)**	**Age range**	**Device**
Barbados	Barbados_1978 ([1])	Barbados	62 (F28; M34)	5.5–11.4	DEDAAS
China	Chengdu_2014 ([2])	Chengdu	33 (F7; M26)	21–28	BrainAmp
	Chongqing_2016 ([3])	Chongqing	235 (F134; M101)	15–26	BrainAmp
Colombia	Colombia_2019	Colombia	21 (F13; M8)	22–45	Neuro scan
Cuba	Cuba_90 ([4])	Cuba90	195 (F98; M97)	5.5–97	Medicid-3M
	Cuba_2003 ([5])	Cuba2003	48 (F28; M20)	5–69	Medicid-4
	Cuba_2004 (?)	Cuba2004	14 -	22–48	?
	CHBMP ([6])	CHBMP	124 (F27; M97)	17–62	Medicid-5
Germany	Germany_2013 ([7])	Germany	178 (F113; M65)	22.5–77.5	BrainAmp
Malaysia	Malaysia_2017	Malaysia	26 (F24; M2)	19–60	ANT Neuro
Russia	Russia_2013 ([8])	Russia	58 (F34; M24)	18–49	nvx136
		same	145 (F70; M75)	16–57	actiCHamp
Switzerland	Bern_1980 ([9])	Bern	44 (F18; M26)	10–16	Nihon Koh
	Zurich_2017 ([10])	Switzerland	165 (F80; M85)	18–90	EGI-256 HC
USA	New York_1970s ([11])	NewYork	230 (F109; M121)	6–80.5	DEDAAS
Total		1564 (F783; M781)			

Table 1: Multi-national EEG norms dataset as described in [12]. It consist of 9 countries, 12 devices and 14 batches.

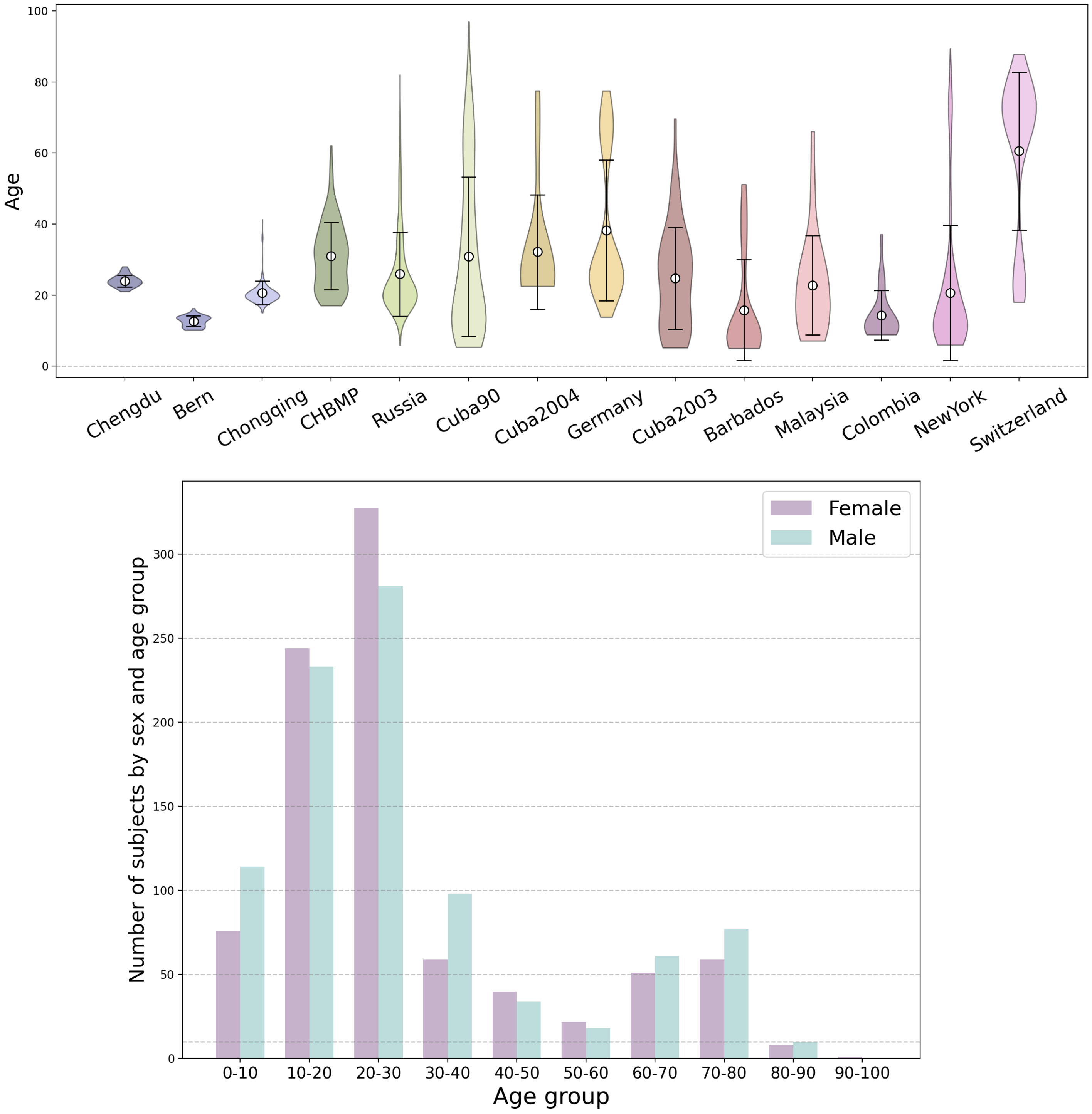

Figure 1: HarMNqEEG dataset demographics. Top Panel: Distribution of individuals in the data set by age and batch. Bottom Panel: Distribution of individuals in the data set by sex and age group. Both figures show that the data set is not uniform in each site or age range.

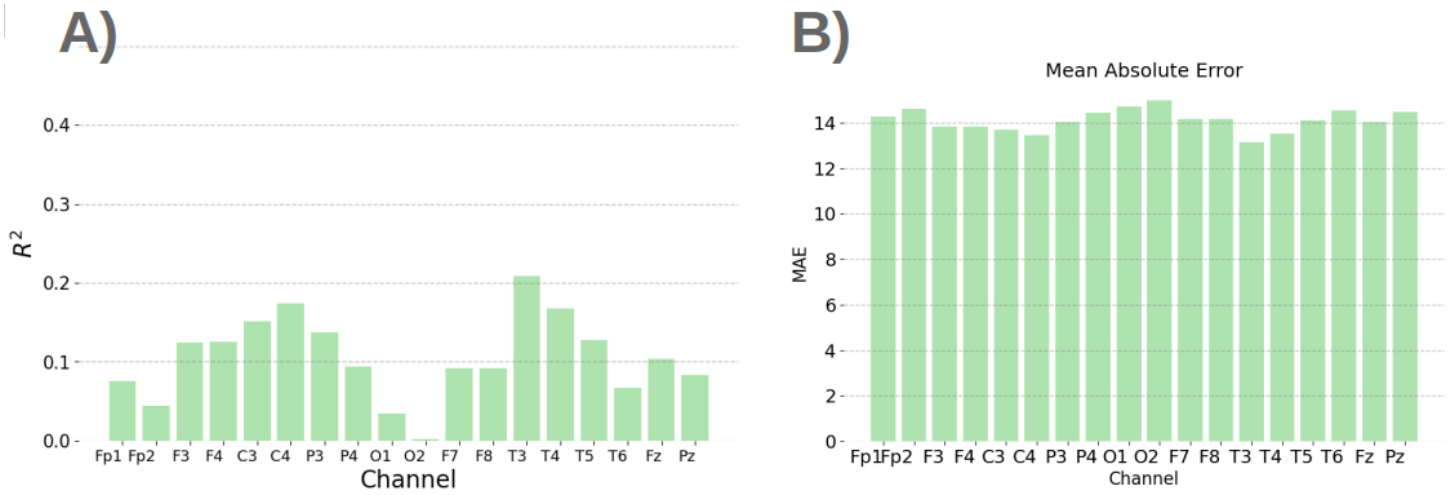

Figure 2: Results for KMER with a linear kernel $f(\cdot)$. **A)** R^2. **B)** Mean Absolute Error.

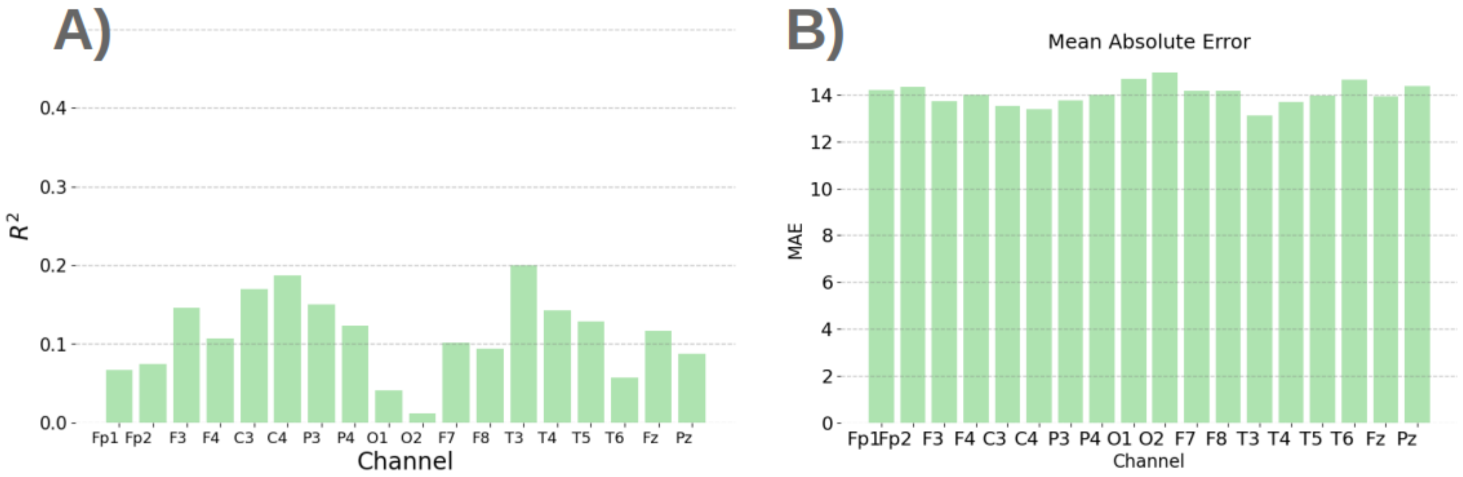

Figure 3: Results for KMER with a polynomial kernel $f(\cdot)$. **A)** R^2. **B)** Mean Absolute Error.

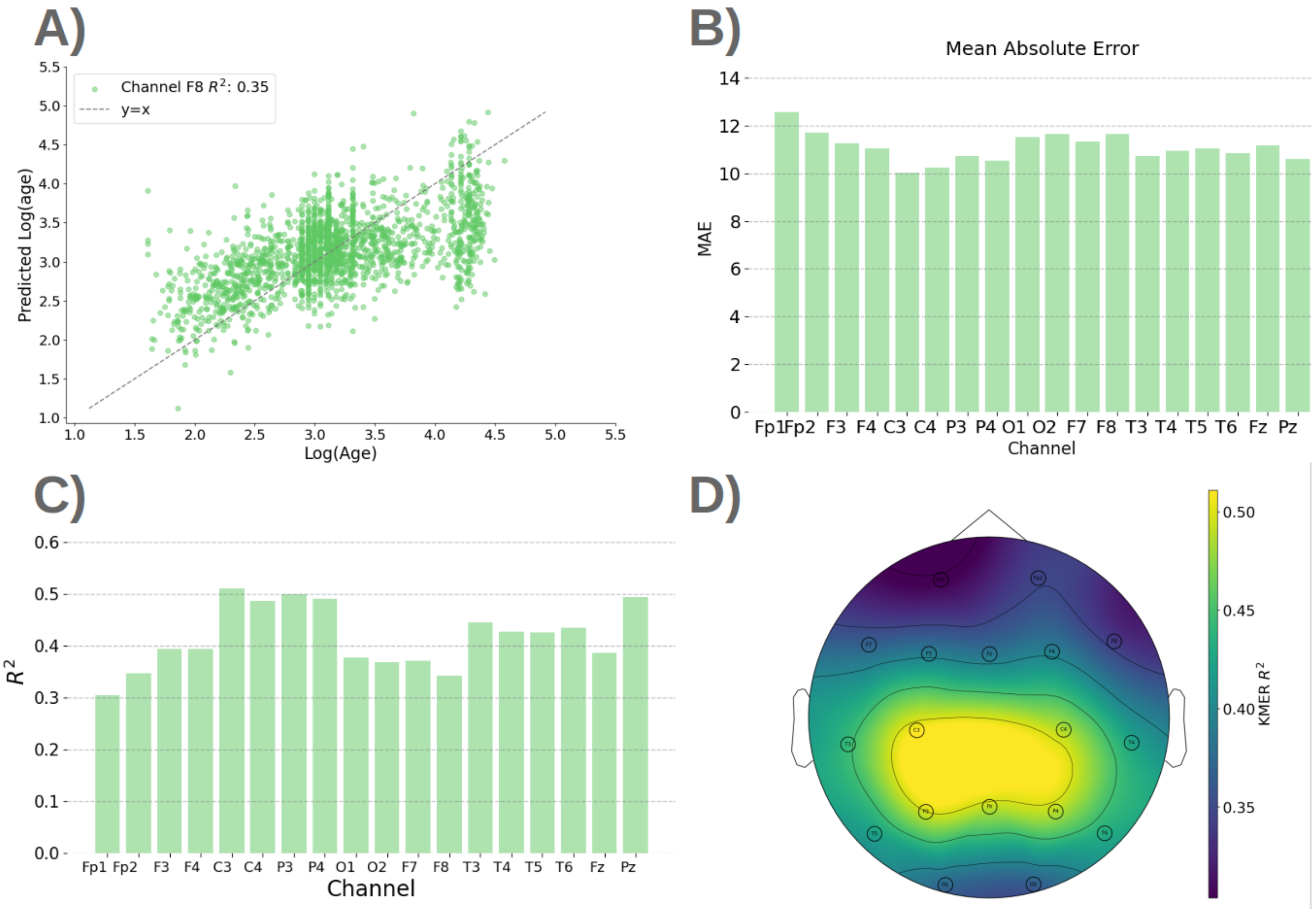

Figure 4: Results for KMER using the logarithmic of age. **A)** Illustration of predicted vs. real age for channel T3. **B)** MAE. **C)** R^2. **C)** R^2 on a topographic map.

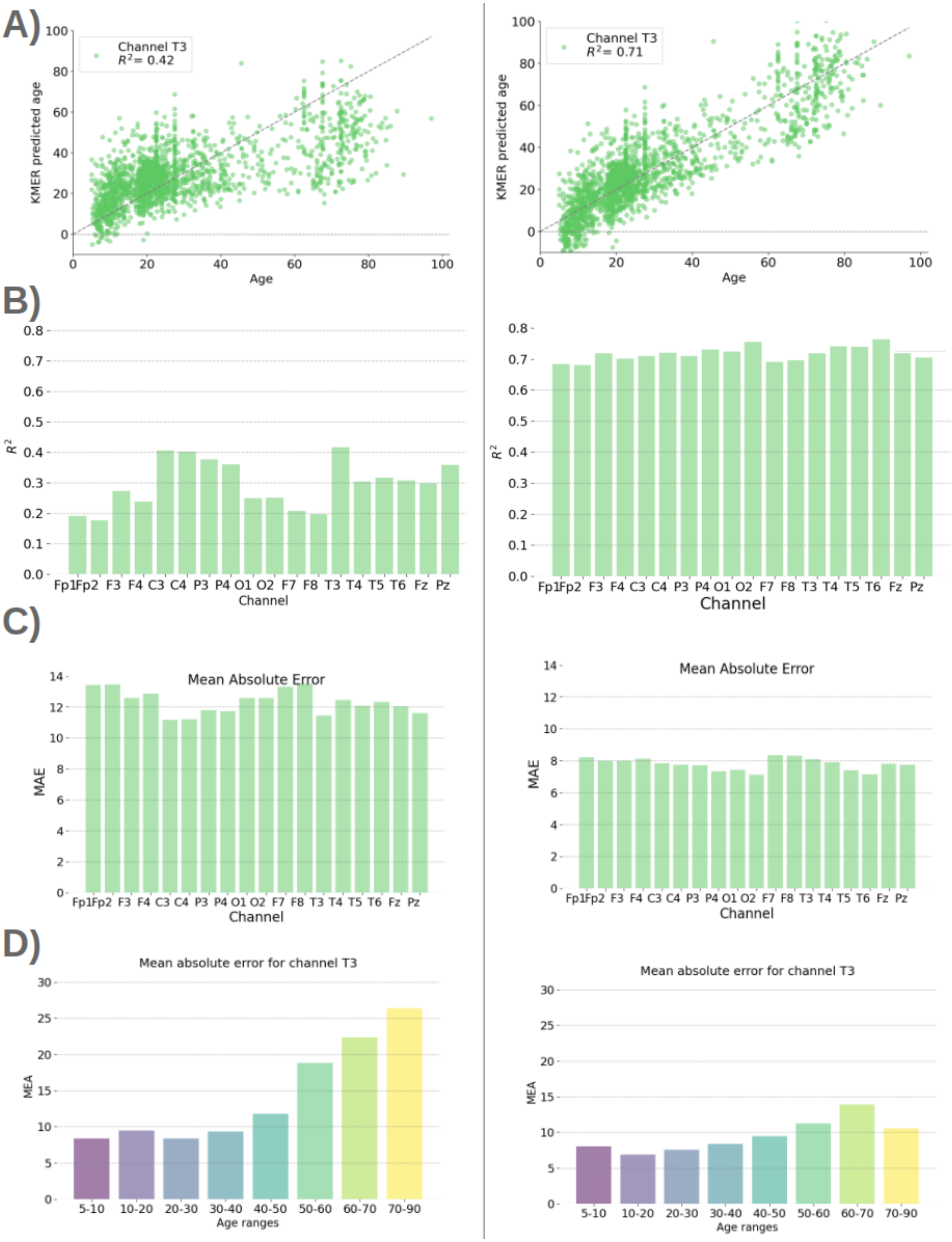

Figure 5: Effect of applying a posthoc bias correction on KMER. Left panels correspond to uncorrected results, and right panels to corrected results. **A)** Illustration of predicted vs. real age for channel T3. **B)** Explained variance R^2 per channel. **C)** MAE per channel. **D)** MAE per age range.

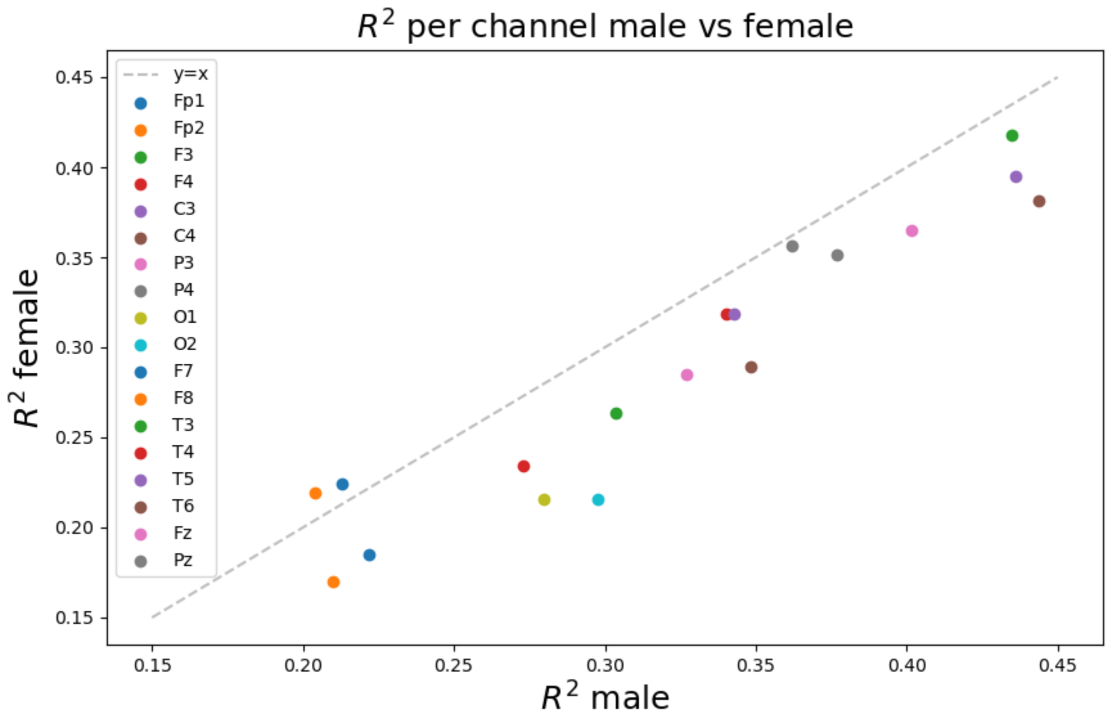

Figure 6: Male vs female accuracies (one point per channel.

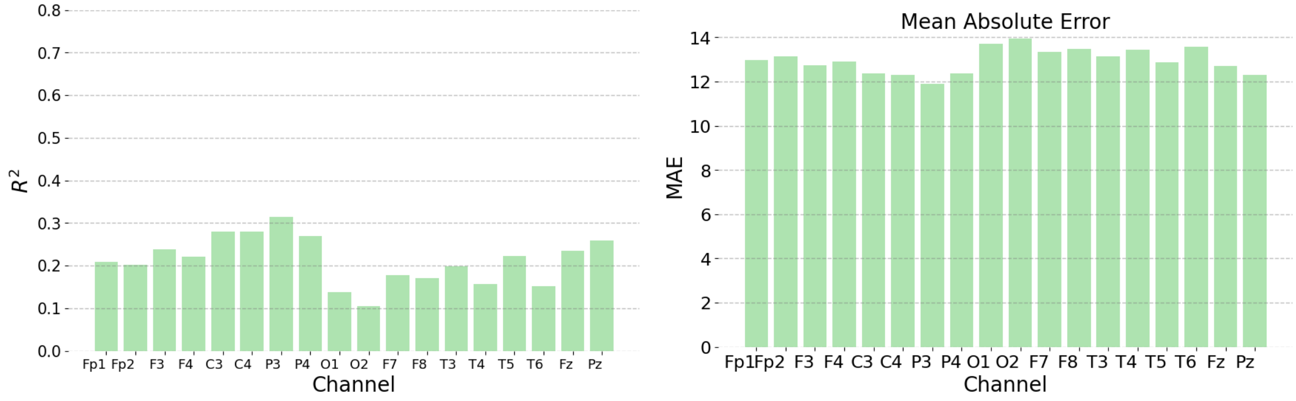

Figure 7: Effect of Normalization. R^2 and MAE for KMER with each bin normalize across subjects (before scaling them to sum to 1) such that the variance is comparable across bins.

References

[1] M. L. Bringas Vega, Y. Guo, Q. Tang, F. A. Razzaq, A. Calzada Reyes, P. Ren, D. Paz Linares, L. Galan Garcia, A. G. Rabinowitz, J. R. Galler, J. Bosch-Bayard, and P. A. Valdes Sosa, "An age-adjusted eeg source classifier accurately detects school-aged barbadian children that had protein energy malnutrition in the first year of life," *Frontiers in Neuroscience*, vol. 13, 2019.

[2] F. Li, T. Liu, F. Wang, H. Li, D. Gong, R. Zhang, Y. Jiang, Y. Tian, D. Guo, D. Yao, and P. Xu, "Relationships between the resting-state network and the p3: Evidence from a scalp eeg study," *Scientific Reports*, vol. 5, p. 15129, Oct 2015.

[3] W. Duan, X. Chen, Y.-J. Wang, W. Zhao, H. Yuan, and X. Lei, "Reproducibility of power spectrum, functional connectivity and network construction in resting-state eeg," *Journal of Neuroscience Methods*, vol. 348, p. 108985, 2021.

[4] J. Bosch-Bayard, L. Galan, E. Aubert Vazquez, T. Virues Alba, and P. A. Valdes-Sosa, "Resting state healthy eeg: The first wave of the cuban normative database," *Frontiers in Neuroscience*, vol. 14, 2020.

[5] G. Hernandez-Gonzalez, M. L. Bringas-Vega, L. Galán-Garcia, J. Bosch-Bayard, Y. Lorenzo-Ceballos, L. Melie-Garcia, L. Valdes-Urrutia, M. Cobas-Ruiz, P. P. A. Valdes-Sosa, and C. H. B. M. P. (CHBMP), "Multimodal quantitative neuroimaging databases and methods: The cuban human brain mapping project," *Clinical EEG and Neuroscience*, vol. 42, no. 3, pp. 149–159, 2011. PMID: 21870466.

[6] P. A. Valdes-Sosa, S. Galan-Garcia, Lidice AU own, L. Valdes-Urrutia, A. C. Evans, and M. J. Valdes-Sosa, "The cuban human brain mapping project, a young and middle age population-based eeg, mri, and cognition dataset," *Scientific Data*, vol. 8, p. 45, Feb 2021.

[7] A. Babayan, M. Erbey, D. Kumral, J. D. Reinelt, A. M. F. Reiter, J. Röbbig, H. L. Schaare, M. Uhlig, A. Anwander, P.-L. Bazin, A. Horstmann, L. Lampe, V. V. Nikulin, H. Okon-Singer, S. Preusser, A. Pampel, C. S. Rohr, J. Sacher, A. Thöne-Otto, S. Trapp, T. Nierhaus, D. Altmann, K. Arelin, M. Blöchl, E. Bongartz, P. Breig, E. Cesnaite, S. Chen, R. Cozatl, S. Czerwonatis, G. Dambrauskaite, M. Dreyer, J. Enders, M. Engelhardt, M. M. Fischer, N. Forschack, J. Golchert, L. Golz, C. A. Guran, S. Hedrich, N. Hentschel, D. I. Hoffmann, J. M. Huntenburg, R. Jost, A. Kosatschek, S. Kunzendorf, H. Lammers, M. E. Lauckner, K. Mahjoory, A. S. Kanaan, N. Mendes, R. Menger, E. Morino, K. Näthe, J. Neubauer, H. Noyan, S. Oligschläger, P. Panczyszyn-Trzewik, D. Poehlchen, N. Putzke, S. Roski, M.-C. Schaller, A. Schieferbein, B. Schlaak, R. Schmidt, K. J. Gorgolewski, H. M. Schmidt, A. Schrimpf, S. Stasch, M. Voss, A. Wiedemann, D. S. Margulies, M. Gaebler, and A. Villringer, "A mind-brain-body dataset of mri, eeg, cognition, emotion, and peripheral physiology in young and old adults," *Scientific Data*, vol. 6, p. 180308, Feb 2019.

[8] R. Ivanov, F. Kazantsev, E. Zavarzin, A. Klimenko, N. Milakhina, Y. G. Matushkin, A. Savostyanov, and S. Lashin, "Icbraindb: An integrated database for finding associations between genetic factors and eeg markers of depressive disorders," *Journal of Personalized Medicine*, vol. 12, no. 1, 2022.

[9] T. Koenig, L. Prichep, D. Lehmann, P. V. Sosa, E. Braeker, H. Kleinlogel, R. Isenhart, and E. John, “Millisecond by millisecond, year by year: Normative eeg microstates and developmental stages,” *NeuroImage*, vol. 16, no. 1, pp. 41–48, 2002.

[10] N. Langer, C. C. von Bastian, H. Wirz, K. Oberauer, and L. Jäncke, “The effects of working memory training on functional brain network efficiency,” *Cortex*, vol. 49, no. 9, pp. 2424–2438, 2013.

[11] H. Ahn, L. Prichep, E. R. John, H. Baird, M. Trepetin, and H. Kaye, “Developmental equations reflect brain dysfunctions,” *Science*, vol. 210, no. 4475, pp. 1259–1262, 1980.

[12] M. Li, Y. Wang, C. Lopez-Naranjo, S. Hu, R. C. G. Reyes, D. Paz-Linares, A. Areces-Gonzalez, A. I. A. Hamid, A. C. Evans, A. N. Savostyanov, A. Calzada-Reyes, A. Villringer, C. A. Tobon-Quintero, D. Garcia-Agustin, D. Yao, L. Dong, E. Aubert-Vazquez, F. Reza, F. A. Razzaq, H. Omar, J. M. Abdullah, J. R. Galler, J. F. Ochoa-Gomez, L. S. Prichep, L. Galan-Garcia, L. Morales-Chacon, M. J. Valdes-Sosa, M. Tröndle, M. F. M. Zulkifly, M. R. B. Abdul Rahman, N. S. Milakhina, N. Langer, P. Rudych, T. Koenig, T. A. Virues-Alba, X. Lei, M. L. Bringas-Vega, J. F. Bosch-Bayard, and P. A. Valdes-Sosa, “Harmonized-multinational qeeg norms (harmnqeeg),” *NeuroImage*, vol. 256, p. 119190, 2022.
